# Development and evaluation of a passive trunk support system for Duchenne muscular dystrophy patients

**DOI:** 10.1186/s12984-018-0353-3

**Published:** 2018-03-14

**Authors:** Mohammad Nauzef Mahmood, Laura H. C. Peeters, Micha Paalman, Gijsbertus J. Verkerke, Idsart Kingma, Jaap H. van Dieën

**Affiliations:** 1 Dept of Human Movement Sciences, Vrije Universiteit, Amsterdam, The Netherlands; 2 Dept of Rahabilitation, Radboud University Medical Center, Nijmegen, The Netherlands; 3 Dept of Physics and Medical Technology, VU University Medical Center, Amsterdam, The Netherlands; 4 Dept of Rehabilitation Medicine, University of Groningen, University Medical Center Groningen, Groningen, The Netherlands; 5 dept of Biomechanical Engineering, University of Twente, Enschede, The Netherlands

## Abstract

**Background:**

Patients with Duchenne muscular dystrophy gradually lose the ability to use different muscles of their body. Consequently, they lose the ability to stabilize their trunk against gravity. This hinders them to effectively perform different daily activities. In this paper, we describe the design, realization and evaluation of a trunk orthosis for these patients that should allow them to move their trunk and maintain stability.

**Method:**

This study aimed to primarily assess the effectiveness of the trunk support system in terms of unloading of trunk muscles, so only healthy participants were recruited for this phase of the study. Measurements were done on 10 healthy participants (23.4±2.07 [M±SD] years old, average body weight 68.42±24.22 [M±SD] kg). The experiment comprised maintaining a constant trunk posture in three different device conditions (control without orthosis and two conditions with different configurations of the orthosis), at four different flexion angles (10°, 20°, 30°, 40°) for each device condition and for two load conditions (with and without stretching the arms). Electromyography (EMG) signals from the trunk muscles were measured to estimate activation levels of the trunk muscles (iliocostalis, longissimus, external oblique and rectus abdominis) and a motion capture system was used to record the movement of the participants during the experiment.

**Results:**

Wearing the orthosis caused reductions in longissimus and iliocostalis activity. The average muscle activity level was 5%–10% of maximum voluntary contraction in the unsupported conditions for those particular muscles. This level was reduced to 3%–9% of maximal voluntary contraction for the supported conditions. No effect on external oblique and rectus abdominis activity was observed. Moreover, no pain or discomfort was reported by any of the participants during the experiment. The results from the current experiment also suggests the necessity of lumber stabilizing systems while using trunk orthosis.

**Conclusion:**

The developed orthosis reduces trunk muscle activation level and provides a solid step for further development of support systems for Duchenne muscular dystrophy patients.

**Trial registration:**

The current study was approved by the medical ethics committee Arnhem-Nijmegen (study number: NL53143.091.15), The Netherlands.

## Background

Neuromuscular disorders (NMD) are characterized by progressive muscle weakening and degradation. There are differences in characteristics among the diseases in the group in terms of severity and progression, but they are all progressively disabling. Duchenne Muscular Dystrophy (DMD) is one of the NMD that has a very significant impact on muscle function and on the quality of life of the patients [1]. It is also one of the most common forms of NMD, affecting approximately 1 in every 5000 live male births [2]. It is caused by a mutation of the dystrophin gene and results in degeneration of skeletal, respiratory and cardiac muscles.

The disease results in loss of walking capabilities around the age of twelve followed by loss of upper body movement capabilities during early teens [3, 4]. Although no cure has been discovered yet to stop or [5] reverse the symptoms, existing treatments including medication and therapy have increased the life expectancy of young men with DMD from 14 years in 1960s to about 30 years currently [6].

Several studies have investigated different aspects of the upper body disability of the patients diagnosed with DMD. While one study among adult patients showed that there is a large variability in upper limb functions in terms of muscle strength and range of motion [7], a survey of 350 participants with DMD (1–35 years) and their caregivers from all over the world, indicated that the most essential activities of daily living (ADL) that DMD patients miss doing due to their limitations are: eating, drinking, using phone or computer, personal hygiene and making physical contact (such as shaking hands) [8]. Furthermore, knowledge gathered from 213 DMD patients suggested participation in school and work related activities have positive effect on the patients [9]. All of these studies indicate that support devices that can compensate for muscle weakness, can provide support for better posture and can help the patients to perform daily tasks without the help of a care giver, which would significantly improve their quality of life. However only a very small percentage of the total population with DMD (around 8.5%) uses assistive devices (mainly arm supports) for such purposes [8].

In recent years, a substantial number of arm supports has been developed for patients with limited movement capabilities [5, 10–12]. However, these systems were developed with a focus on arm functionality only. Consequently, except for one, they were designed to be attached to the wheelchair and the movement of the trunk during the performance of ADL was not taken into consideration during development. To use these assistive devices, the trunk is more or less rigidly fastened to the wheelchair back and this might result in faster degradation of trunk muscles due to a lack of usage and in spinal deformation due to asymmetrical seating [13]. Furthermore, fastening the trunk to the back of the wheelchair also limits the workspace area of the patients. As a result, their performance of ADL is also limited and the movements during these tasks are not comparable to the patterns in healthy subjects.

To avoid these problems, assistive devices for the trunk are desirable. These devices should allow the trunk to move through the range that is used during ADL in healthy persons. Furthermore, an adaptive trunk support device might enable the patients to move their trunk even when they would have very limited force generating capacity in their trunk muscles. Moreover, the device should be usable while the user is using a wheelchair, preferably without requiring modification of the wheelchair.

The aim of the current study was to develop a wearable passive trunk support system and to test its effect on muscle activation during standardized trunk flexion and reaching tasks in healthy participants. The arguments behind selecting healthy participants for the preliminary test were twofold. Firstly, it is inherently safer to perform initial testing of the device in healthy participants. Secondly, if muscle activation in healthy participants would be substantially reduced when wearing the device, this would reveal its potential in assisting patients. This system can then further be tested DMD participants while limiting the risk of harm.

## Methods

### Design boundaries

At the first stage of the development, DMD patients and physicians were interviewed to discuss the desired functionality and appearance of a trunk support system. There are several trunk support systems [14–17] currently available which were developed keeping mainly 3 types of target groups in mind: users doing heavy lifting or working long durations in inclined position while standing, users with low back pain and users undergoing rehabilitation after stroke or spinal injury. None of these devices are directly suitable for DMD population because they do not meet the following requirements for this population: designed for a seated position, mounted on wheel chair, possibility to combine with other support systems, modifying the support level in short term based on daily tiredness and in long term along with disease progression, inconspicuousness. As a result, there is no trunk support system currently being used by patients with DMD. In order to achieve the above-mentioned requirements, which were found through the interview sessions with patients and physicians, several ideas were created and eventually the best one was chosen through a standard design approach [18].

### Features of the orthosis

**Fig. 1:**
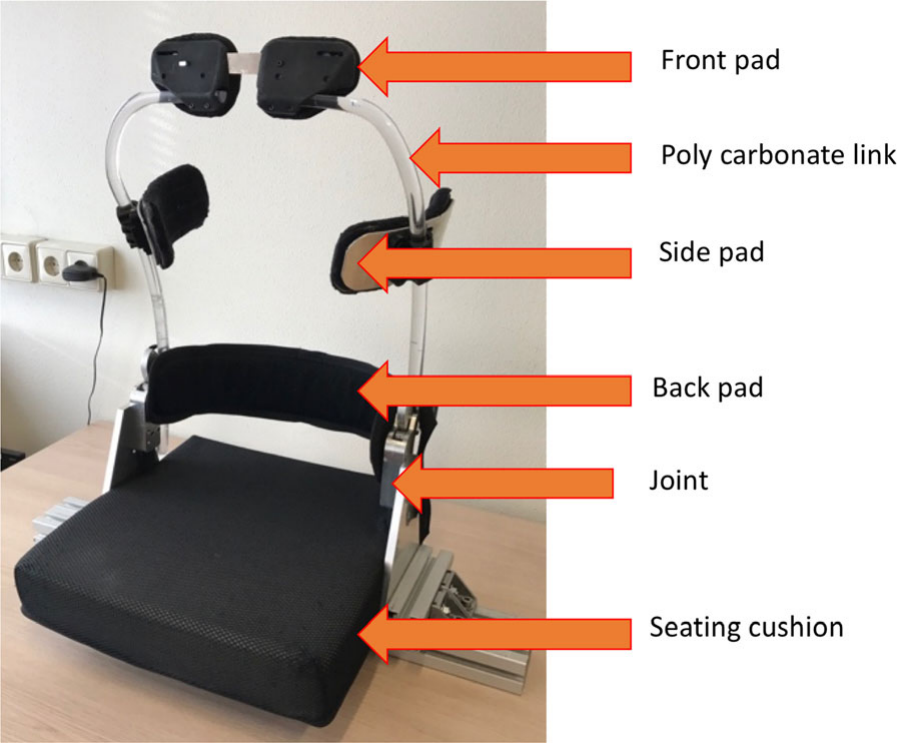
The prototype used in the present study with different parts

**Fig. 2:**
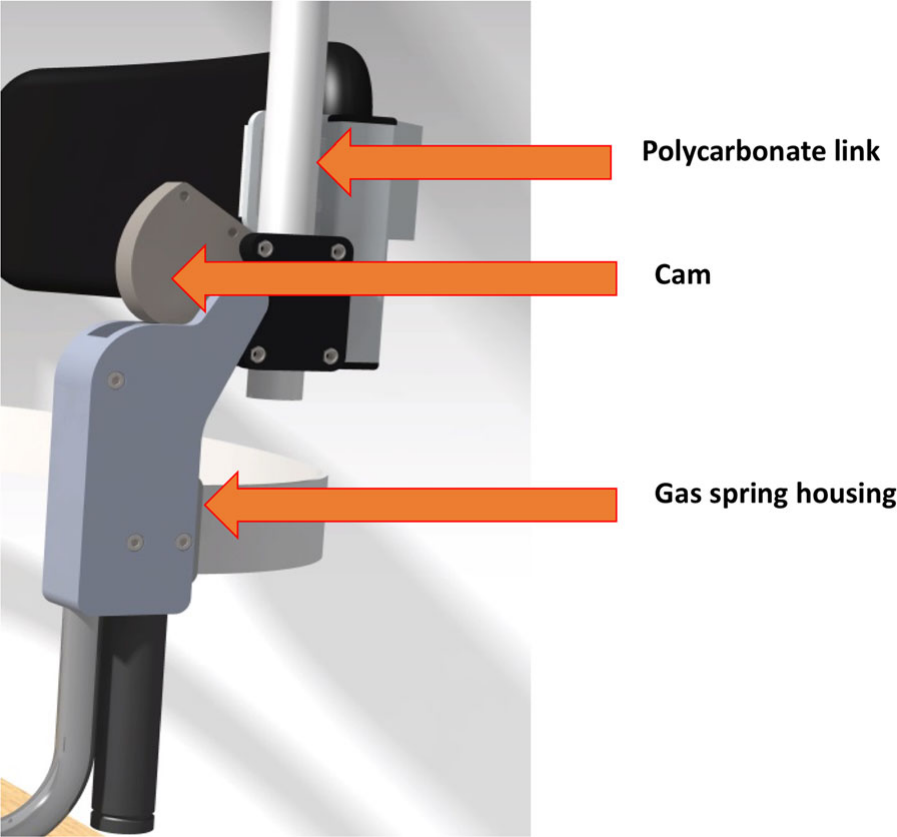
The passive support mechanism

**Fig. 3:**
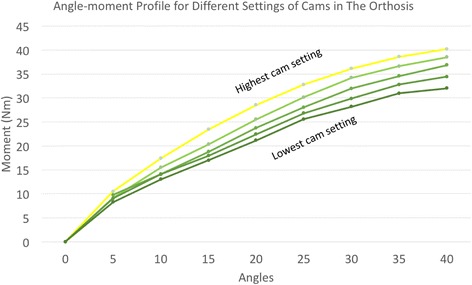
Angle-moment curve for different cam settings of the orthosis

The orthosis has five interface areas with the body: a front pad placed on the sternum, two adjustable side pads at the thoracic level to provide stability, one back pad for lower lumbar support and a cushion on the base plate for sitting (Fig. 1). For gravity compensation, the rotating joint contains a combination of a gas spring in pre-tension with a custom-made cam to push against the gas spring piston (Fig. 2). The technology behind this joint was developed by InteSpring B.V.(The Netherlands) (Patent No: WO/2015/041532) [19]. The shape of the cams can be modified to generate an individualized non-linear gravity compensating moment profile. There are polycarbonate (PC) links on both sides of the trunk connecting the front pad with the rotating joint. The polycarbonate links also act as a spring due to their deformability. During flexion, the gravitational moment of the trunk deforms the links and shortens the gas spring at the rotating joints of the device. This spring energy is returned to the user during the extension enabling him/her to easily lift the upper body with less back muscle activity. The range of movement of the orthosis is 0–40° of flexion. Several sets of cams were made to be used in the current experiment to adjust the balancing level according to each participant’s body dimensions. Thus, the orthosis could be modified to maximally provide between 32 Nm (for the lowest cam setting) to 40 Nm (for the highest cam setting) of torque to the user at 40 degree flexion. This range was selected based on the upper body torque measurements during the design phase of the orthosis which included 10 healthy participants with different body weights ranging from 65Kg to 85 Kg. The different angle-moment profiles for different cam settings are shown in Fig. 3.

The side pads and the length of the PC links can be adjusted for better fitting with the user’s body. By design the orthosis supports the motion in the plane of flexion-extension (sagittal plane) only. Lateral bending is not allowed, because of the presence of the side pad, axial twisting is possible to some extent, but is not supported by the prototype.

### Participants

Ten healthy adult males (23.4±2.07 [M±SD] years old, average body weight 68.42±24.22 [M±SD] kg) participated in the experiment. The exclusion criteria were: injury/ pain/ pathologies affecting the neuromuscular system, communication problems or severe sensory impairment. Only male participants were chosen as DMD only affects the male population. The experimental procedure was approved by the medical ethics committee Arnhem-Nijmegen (study number: NL53143.091.15), The Netherlands. Written informed consent were obtained from all the participants prior to their participation in the experiment.

### Study protocol

First, maximum voluntary contractions (MVC) were measured for the iliocostalis, longissimus, external oblique and rectus abdominis muscles. For this step, the participants were asked to lie horizontally on a platform keeping upper half of their body outside the platform with their legs strapped to the platform. From that position, they were asked to press against the hands of a researcher with their trunk with maximum possible force. The participant’s orientation and the position of the hands of the researcher were changed to measure the MVC of the different trunk muscles.

**Fig. 4:**
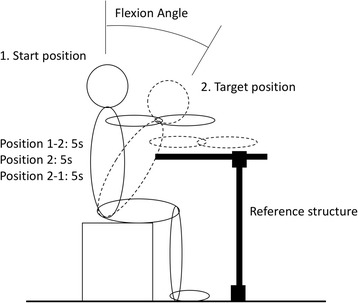
The experimental steps in different experimental condition

Next, the participants were asked to sit in an upright position. A kinematic calibration trial was recorded in the upright seated position with both hands on the knees. This upright position was taken as the reference position for the other trials and for the kinematic measurements. Then, from the reference position, they were asked to slowly flex their trunk to predefined inclination angles (10, 20, 30 or 40 degrees), using a reference structure that was adapted to the individual position of a point on the chest at the required angle. The participants were asked to incline forward until they touched the reference structure with their body, then they were given feedback to adjust their posture based on the reading from the accelerometers while touching the reference. Once the desired posture was achieved, they were then asked to hold their inclined posture at that position for 5 s, while gently touching the reference structure, and then slowly extend the trunk to the initial position (Fig. 4). The timing of the movement was controlled by a metronome. Each angle was measured in two load conditions, with three device conditions, and with two repetitions. The load conditions were arms put on the shoulder without stretching and arms stretched horizontally forward. The reason for including a stretched arm posture was to observe the effect of added torque on the trunk, similar to when reaching for an object. After one set of measurements (2 load conditions and 2 repetitions), the reference point was set to one of the other target trunk angles. As the muscle activity level depends both on load on the muscle and muscle fibre length, the participants were given feedback on the pelvic inclination and trunk inclination using real time information from accelerometers attached to the sacrum and T6 vertebra, in order to make adjustment to the posture in the flexed position. Another reading was taken at the target inclined position by using two accelerometers to record pelvic and thoracic inclination. Then the reference structure was set at this position. In the following measurement trials, when the participants were touching the reference structure, they were provided with feedback to adjust their pelvic and thoracic inclination based on the recorded accelerometer values obtained during the calibration if the deviation in posture in the measurement trial was more than 5 degrees from the posture in the calibrated trial. This ensured that the participants had a similar posture for similar inclined angles in different experimental conditions and the measured muscle activity levels would then only be dependent on muscle forces and not on the muscle fibre length.

**Fig. 5:**
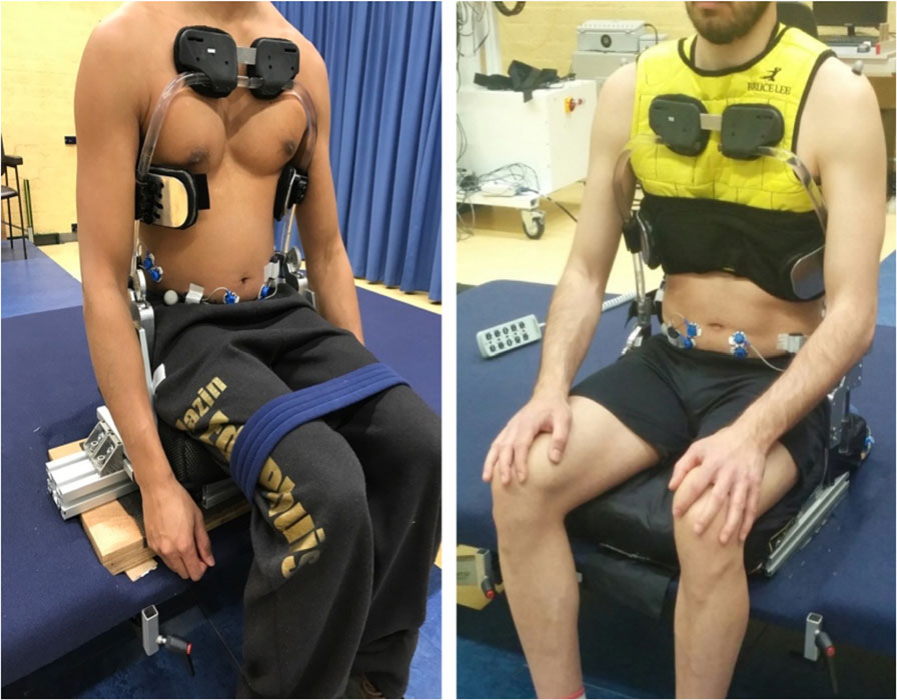
Different configurations of the orthosis (left: orthosis without vest, right: orthosis with vest)

The same movements were measured in three device conditions: a control and two conditions with the orthosis: one without and one with an additional tight vest (Fig. 5). The vest does not affect the functionality of the mechanism of the orthosis, but distributes the contact force and makes the interface with the orthosis better fitted with user’s body. The order of the device conditions was changed among the participants to prevent bias. Before starting the experiment, the orthosis was configured according to the participant’s body weight by installing the cams appropriate for the participant’s weight.

A questionnaire, consisting of questions regarding functionality, comfort and compatibility (each factor was scored with a range from 1 to 10) of the orthosis was used to obtain feedback from the participants before and after the experiment to observe qualitative effects of the orthosis.

### Measurements

**Fig. 6:**
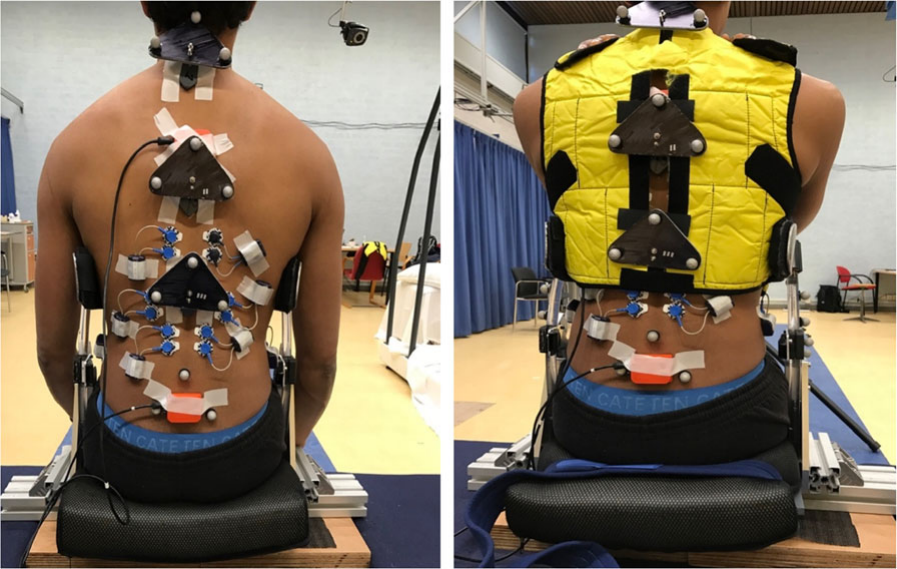
Placement of the sensors on the back of the participants and on the orthosis

The placements of electromyography (EMG) sensors, marker clusters for movement analysis and inertial sensors are shown in Fig. 6. The muscle activity was measured by using five pairs of sEMG electrodes (AG-AGCL, ARBO EMG electrodes, Tyco Healthcare, Neustadt, Germany) (Zerowire EMG, Aurion, Italy), placed over the thoracic part of longissimus (TLO), lumbar part of longissimus (LLO), iliocostalis (IC), external oblique (EO) and rectus abdominis (RA). The position of the electrodes was based on SENIAM guidelines [20]. The electrodes had 24 mm diameter and inter-electrode distance was kept at 20 mm. They were attached to the skin after shaving (when needed) and cleaning with body scrub. Two accelerometers (MTx, Xsens Technologies, Netherlands) were placed on the back of the participants (one on the sacrum and one on at T6 level).

To obtain a more detailed indication of spine curvature and deformation of the orthosis, a three-dimensional motion capture system (Vicon, Oxford metrics, UK) was used to record the trunk posture during the trials with a sampling rate of 100 Hz. Twenty-five reflective markers were placed at the spine (marker clusters at C7, T6 and T12 level), the pelvis and on both sides of the orthosis (see Fig. 6). Markers on the orthosis were used to measure the position of the deformed PC links of the orthosis during each experimental step.

### Data analysis

#### EMG analysis

The EMG signals recorded (sample frequency 1000 samples/s) during the experiment were filtered using a 2^nd^order bi-directional band-pass filter (10–400 Hz), a high pass filter (cut-off frequency 30 Hz, to remove contamination from the electrocardiogram [21]), a band-stop filter (49.5–50.5 Hz, to remove hum artefacts) and subsequently rectified. For this experiment, EMG signals from the static part (when the participants were holding their flexed posture touching the reference structure) of each experimental step were selected for analysis, to observe the effect of the orthosis on muscle activation, while excluding dynamic components of the EMG signal. Then the signals were normalized with respect to MVC and the mean values were obtained for each muscle in each experimental condition.

#### Kinematic analysis

Three-dimensional angles of the trunk segments and pelvis were calculated for the static inclined position based on the marker data obtained from the motion capture system with use of Matlab 8.4.0 (R2014b). An X-Y-Z (flexion - lateral bending – rotation) Cardan angle rotation sequence was used to calculate the inclination of each segment in each trial [22, 23]. The pelvic angles were expressed relative to the world coordinate system and the angles of the trunk segments relative to the more caudal segment. The relative flexion angle of all trunk segments and the pelvic inclination were added for each trial to obtain the overall inclination of the trunk. The trunk inclination angle, measured while the participant was sitting upright during the calibration trial, was used to reference the angles during the other trials for each individual participant.

#### Statistics

Average kinematic and EMG values for TLO, LLO, IC, EO and RA were acquired for 10 participants for the 5 s static inclined position. Mean values of kinematic angles and mean normalized EMG values were calculated from two trials per condition for further analysis. These values were used in SPSS24 (SPSS Inc. Chicago, IL, USA) for statistical analysis by repeated measures analysis of variance (ANOVA). Three device conditions (no orthosis, orthosis without vest and orthosis with vest) and four inclination angles (10, 20, 30 and 40 degrees) were used as factors. This was followed by Bonferroni-corrected multiple pairwise comparisons in case of significant effects of device condition. The significance level for the statistical analysis was set at *p* < 0.05.

## Results

### Muscle activation level

**Fig. 7:**
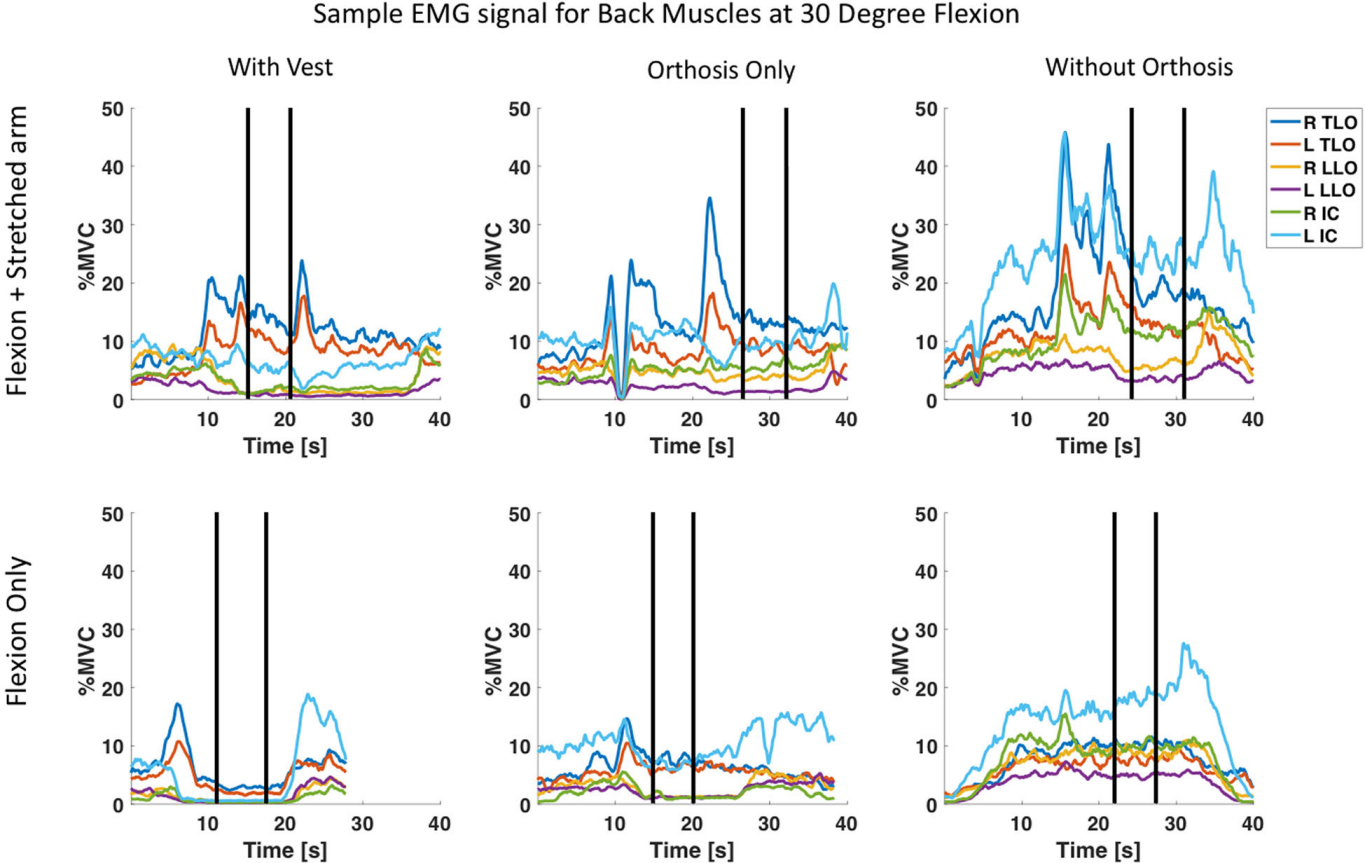
Sample EMG wave from for one participant for trunk muscles on right side (R TLO, R LLO and R IC) and trunk muscles on left side (L TLO, L LLO, L IC) at 30 degree flexion for different device conditions. The time window between the two vertical lines is the static phase which was used for further calculation

**Fig. 8:**
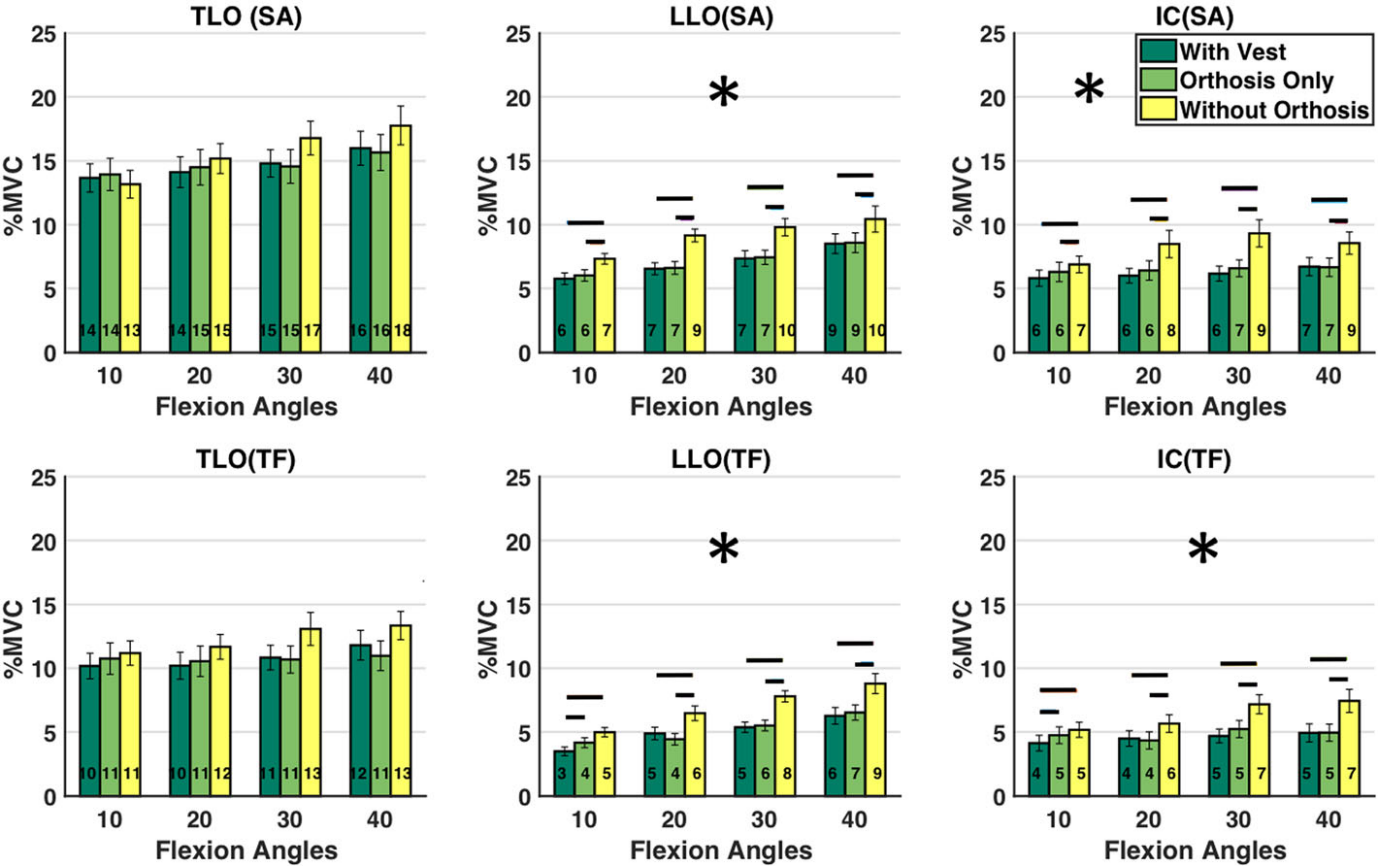
Average muscle activation levels for longissimus and iliocostalis at different flexion angles. Upper row shows flexion with stretched arms (SA) and lower row shows trunk flexion only (TF). Horizontal lines indicate significant differences (*p* < 0.05) between the device conditions below the end point of the lines. *For this [muscle / SA or TF] condition pairwise comparisons were performed on averages over flexion angles (see Table 2) as no interactions between device condition and trunk inclination were found

A sample set of EMG envelopes from TLO, LLO and IC from one of the participants at 30 degree trunk flexion in different device conditions is shown in Fig. 7. A significant main effect of angle on activity of lumbar back muscles (LLO and IC) and for the thoracic muscle (TLO) was found, but not on abdominal muscle activity (Table 1, Fig. 8). Note however that, without the orthosis and without stretching the arms, muscle activation of the back muscles was already quite substantial in the 10 degrees target trunk angle (11, 5 and 5% MVC for the TLO LLO and IC, respectively) and increased by only 2–4% MVC at 40 target trunk angle. Abdominal muscle activity was low across all conditions with average EMG amplitudes below 4 %MVC and was not affected significantly by the different device conditions.

**Table 1:** *P*-values of two-way repeated measures ANOVA

	Trunk Flexion only		Trunk Flexion + Stretched Arm			Trunk Flexion only		Trunk Flexion + Stretched Arm		Device Cond.* Angle (Flex Only)	Device Cond.* Angle (Flex + Arm)
Device Cond.	Mean	*P*	Mean	*P*	Angles Cond.	Mean	*P*	Mean	*P*		
All average											
With Orthosis + Vest	6.9	**< 0.001**	9.4	**0.002**	flexion 10	6.8	**0.003**	8.7	**0.006**	0.122	0.067
With Orthosis Only	7.1		9.6		flexion 20	7.1		9.9			
Without Orthosis	8.9		11.4		flexion 30	8.1		10.6			
					flexion 40	8.5		11.2			
TLO											
With Orthosis + Vest	10.9	0.064	15.1	0.174	flexion 10	11.2	0.151	13.9	**0.007**	0.468	**0.001**
With Orthosis Only	11.3		15.2		flexion 20	10.9		15.1			
Without Orthosis	12.5		16.3		flexion 30	11.8		15.9			
					flexion 40	12.4		17.0			
LLO											
With Orthosis + Vest	4.9	**< 0.001**	6.8	**< 0.001**	flexion 10	4.3	**0.001**	6.2	**0.039**	0.253	0.287
With Orthosis Only	5.3		7.0		flexion 20	5.0		7.2			
Without Orthosis	6.8		8.7		flexion 30	6.1		7.9			
					flexion 40	7.0		8.8			
IC											
With Orthosis + Vest	5.0	**0.005**	6.6	**0.015**	flexion 10	5.3	**< 0.001**	6.9	0.241	0.21	0.153
With Orthosis Only	5.6		7.0		flexion 20	5.3		7.6			
Without Orthosis	7.0		9.1		flexion 30	6.3		7.9			
					flexion 40	6.7		7.8			
EO											
With Orthosis + Vest	1.7	0.199	2.2	0.66	flexion 10	1.6	0.453	1.9	0.226	0.766	0.211
With Orthosis Only	1.6		2.4		flexion 20	1.5		1.9			
Without Orthosis	1.5		2.4		flexion 30	1.5		2.4			
					flexion 40	1.8		3.1			
RA											
With Orthosis + Vest	0.6	0.744	0.6	0.358	flexion 10	0.5	0.227	0.6	0.263	0.419	0.282
With Orthosis Only	0.6		0.6		flexion 20	0.6		0.6			
Without Orthosis	0.6		0.7		flexion 30	0.5		0.7			
					flexion 40	0.8		0.7			

Data in bold are statistically significant, statistical significance was set at *P* < 0.05

Calculation was done with device condition and flexion angle as factors and average muscle activation as outcome measure

Separate ANOVA’s were performed for tests with the arms hanging down (Flex only) and the arms stretched forwards

The ‘*’ represents the interaction among device conditions and angles in repeated measures ANOVA

A significant main effect of device condition on activity of the lumbar back muscles (LLO and IC) was found, both with and without stretching the arms (all *p* < 0.02; see Table 1; Fig. 8). The reduction of activity of LLO and IC when wearing the orthosis (with or without the vest) ranged between 1% - 3% of MVC, compared to the unsupported condition, over inclination angles and load conditions (Fig. 8). The thoracic back muscle (TLO) and abdominal muscles did not show an effect of device condition, although a non-significant tendency (*p* = 0.064) was found for TLO.

Averaged over trunk flexion angles and device conditions, the activation level of the TLO, RA and EO was 15.5±4.08 [M±SD], 2.3±1.3 [M±SD] and 0.6±0.37 [M±SD] of %MVC respectively for the stretched arm condition and 11.6±3.84 [M±SD], 1.6±0.70 [M±SD] and 0.6±0.37 [M±SD] of %MVC respectively for the condition without stretched arms.

No interaction among device conditions and angles was observed for any of the muscles during the repeated measures ANOVA. Follow up post hoc tests (Bonferroni correction) for the main effect of device conditions showed no significant differences in muscle activation between the orthosis with vest condition and the orthosis without vest condition (Table 2).

**Table 2:** Multiple Bonferroni-corrected pairwise comparisons between device conditions for muscles that showed a main effect of device condition in the repeated measures ANOVA

Pairs	Flex Only	Flex + Stretched Arms		
	LLO	IC	LLO	IC
Without orthosis: with orthosis+vest	**0.001**	**0.033**	**0.002**	0.065
Without orthosis: with orthosis only	**0.004**	**0.008**	**0.008**	**0.035**
With orthosis+vest: with orthosis only	0.192	0.589	0.584	0.615

Data in bold are statistically significant, statistical significance was set at *P* < 0.05

### Kinematic analysis

**Fig. 9:**
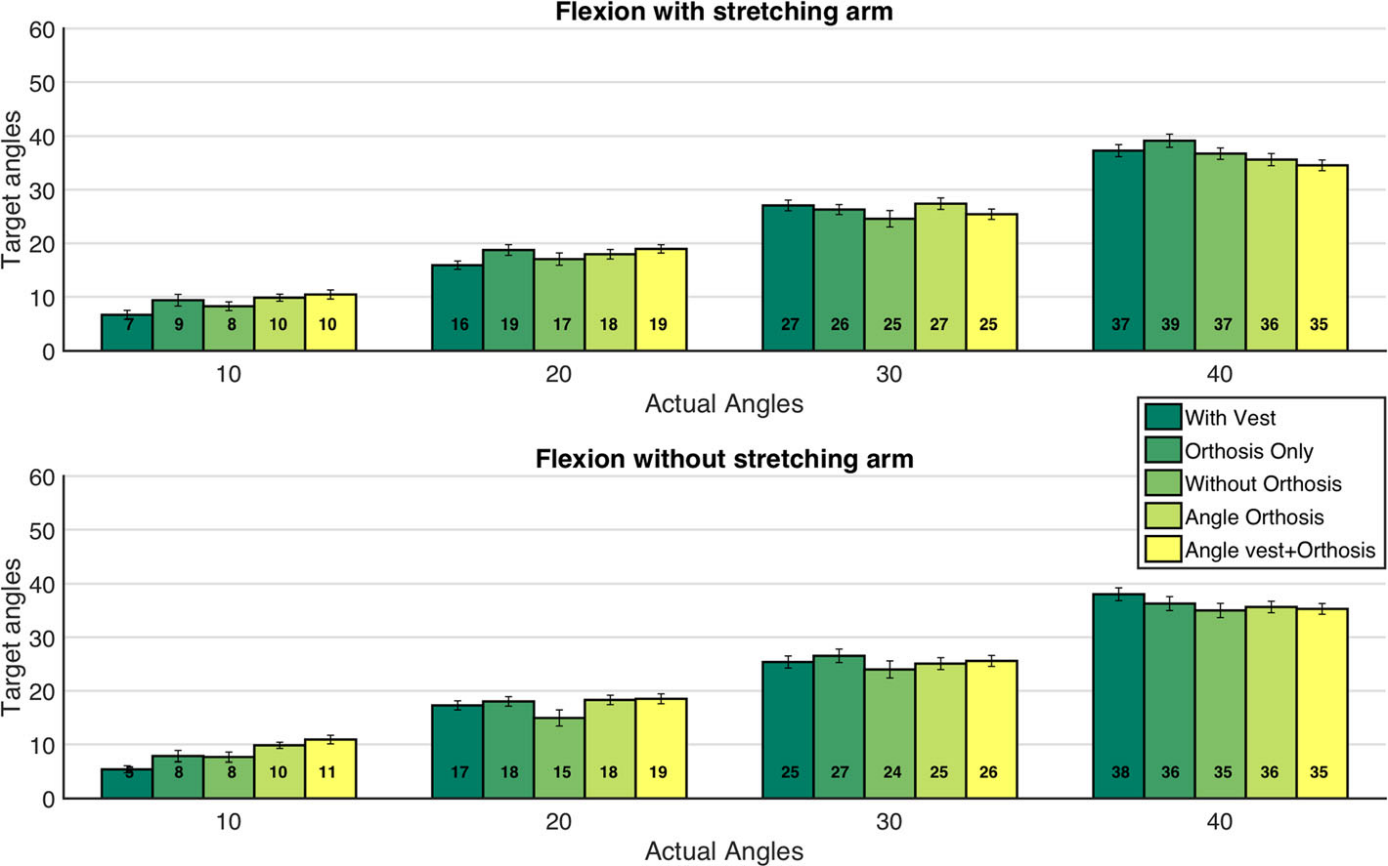
Average actual trunk inclination angles (Pelvic inclination + lumber flexion + lower thoracic flexion) for different experimental conditions. 19 out of total 480 trials were excluded from this analysis as the VICON markers were not visible and the inclination angles could not be calculated

The repeated measures analysis of variance showed no significant main effect (Fig. 9) of device condition (*p* = 0.160), nor a device and trunk inclination angle interaction (*p* = 0.538). The average value of actual trunk inclination angles over all device conditions and load conditions for 10, 20, 30 and 40 degrees of target angles were 6.51±3.93 [M±SD], 18.06±3.97 [M±SD], 26.63±5.07 [M±SD] and 36.706±5.34 [M±SD] degrees respectively.

### Questionnaire

Among 10 participants none reported pain or discomfort while using the orthosis.

## Discussion

The aim of this study was to observe the effect of a novel orthosis on trunk muscle activation in healthy subjects. The reduction in trunk muscle activity achieved with the prototype ranged between 1%–3% MVC. As no previous studies have been performed on the effect of a trunk balancing system for DMD patients, the results from the current study can only be compared with studies done on trunk orthoses developed for the treatment of low back pain or spinal deformities. The reduction in muscle activity in the current study are in good agreement with such studies. A study done on the efficacy of a trunk orthosis during static standing in elderly people reported a decrease of 1–2% MVC for the erector spinae [24]. Another study on postural control tasks such as seating on an unstable platform, showed a 1–2% MVC reduction for thoracic and lumber erector spinae muscles and no significant effect on the abdominal muscle activity [25].

A key question regarding the outcome of the current study is, whether a reduction of 1–3% MVC can be considered to be a substantial support provided by the orthosis to the user. While no straightforward answer can be provided, it is interesting to note that an experimental study showed that [26]. So, a small decrease in %MVC can be equivalent to a large reduction in required weight bearing effort. Furthermore, static muscular contractions can potentially result in fatigue and fatigue related pain if sustained for a long time even at very low levels of muscle activation (5% or higher) [27]. The orthosis reduces the activity level near or below this range. Moreover, DMD patients have weak trunk muscles, so their muscle activation level and the reduction by the orthosis will likely be larger than in healthy control subjects. The reason behind such higher level of remaining activity, is probably related to the need to stabilize the trunk, as it is known that the spine is an unstable structure that buckles at small loads in the absence of muscle activity [28]. The stiff thoracic vest in the present study did not decrease muscle activity. Possibly, a stabilizing lumbar orthosis is needed to achieve a further reduction of muscle activity. This is only applicable for the back muscles. As the abdominal muscles were consistently lower across all experimental conditions, it can be said that the use of the current trunk orthosis does not require the user to push against or work against the orthosis.

The current experiment indicates that the orthosis maintains its effect on back muscle activity even when the load on the trunk muscle increases, i.e. by stretching the arms, making it suitable for daily tasks. The effect of the orthosis however would change when heavy objects are lifted. But as the orthosis is aimed for DMD patients, such heavy lifting tasks are outside the user requirements.

As muscle activation level depends both on muscle load and fibre length, for the success of the current study it was crucial that the participants adopted similar postures for each set of measurements. In general, as the overall trunk inclination angles did not vary significantly over device conditions, it can be argued that the reduction of muscle activity level observed is caused by the use of orthosis and not by postural changes.

The effect of the orthosis may have been limited by mechanical losses in the system, failing to follow the postural instructions properly, and moments applied to the trunk due to changes in head orientation. First, as with any mechanical system, the developed orthosis introduces friction during each movement cycle. This was measured separately by integrating a force sensor at the front pads location of the orthosis and then measuring the moment required to bend the orthosis over many repeated cycles. We observed from this test that the frictional loss ranged between 5%–8%. As a result, the orthosis produces higher moments while bending forward than when in static positions and when bending back. Second, deviations from target angles (10–15 degrees) were seen in 3 participants during some trials, indicating that not all subjects could repeat the postures perfectly over different conditions. However, these deviations did not result in significant postural differences between different device conditions. So, these deviations were not systematic and therefore did not bias our findings. Third, no instructions were given to the participants regarding the orientation of the head during the experiment. The inclination of the head during trials can affect trunk muscle activation during static trunk inclination. As no markers were put on the head of the participants, precise repetition of head inclination could not be ensured. Therefore, the variability of the head orientation may have caused variation in effectivity of the orthosis across subjects.

The following study limitations should be considered. Orientation of the trunk segments was only considered in sagittal plane and not in coronal and transverse planes. This is because the orthosis provides passive support only in sagittal plane. Another limitation of the present study is that we did not measure deep muscles as this is not possible with surface EMG. In general, more reduction of activation is expected for the muscles that are most active during a particular task and are also supported by the orthosis during that particular task [29]. It can be argued that the deeper trunk muscles might also benefit from the orthosis similar to the bigger and superficial trunk muscles measured during the current experiment, but this may not be true for muscles that are largely stabilizing [30]. Although some familiarization with several movements with the orthosis was provided to the users and no pain was reported, it is still possible that some discomfort experienced while using the orthosis may have increased muscle activity levels. The frictional effect in the orthosis was measured separately and not while the participants were performing in the experiment. So, it is not possible to indicate exactly how much friction was present trial. Reduction of frictional loss could increase the effect of the orthosis to some extent. The support provided was not customized for each individual participant, instead predefined moment profiles were selected but scaled based on the body mass of the participants. The use of a generalized moment profile instead of custom made moment profile for each individual participant has introduced some balancing error which limits the effectiveness of the orthosis.

It is still unknown to what extent the trunk muscles are active in case of DMD patients, to what extent they can be reduced and whether they would be affected similarly by the orthosis as in healthy participants. Future studies could investigate such effects. There is a plan to evaluate the current orthosis with DMD patients in near future. If the results from the next experiment presents similar or better advantages for the DMD participants, then the orthosis could be provided to the patients for daily usage. Albeit that additional postural support might be needed to allow for further reduction of required muscle forces in weak patients.

## Conclusion

To conclude, the present study has shown that the orthosis developed to support the trunk of DMD patients can reduce trunk muscle activity levels of the longissimus and iliocostalis muscles at different inclined positions of the trunk and in different load conditions (with and without stretched arms) and does not cause any discomfort or pain to the user. Moreover, the current study indicates that activity of abdominal muscles does not increase significantly when using such trunk orthosis. The knowledges obtained from this study will help further development of the current orthosis to make it more suitable for the target user group in particular and any kind of trunk orthosis or exoskeleton system in general. The prototype presented in the current study provides a step towards helping DMD patients to increase their independence in activities of daily life.

## Abbreviations

ADL: Activities of daily living
ANOVA: Analysis of varience
DMD: Duchenne muscular dystrophy
EMG: Electromyography
EO: External Oblique
IC: Iliocostalis
LLO: Lumber level of Longissimus
M: Mean
MVC: Maximum voluntary contraction
NMD: Neuromuscular disorders
PC: Polycarbonate
RA: Rectus abdominis
SD: Standard Deviation
TLO: Thoracic level of Longissimus
